# Clinical presentation and early predictors of progression to dilated cardiomyopathy in children with acute myocarditis

**DOI:** 10.3389/fped.2025.1616751

**Published:** 2025-07-03

**Authors:** Liu Luo, Yanyun Huang, Xiaoyu Qiao, Yusheng Pang

**Affiliations:** Department of Pediatrics, The First Affiliated Hospital of Guangxi Medical University, Nanning, China

**Keywords:** acute myocarditis, children, dilated cardiomyopathy, prognosis, follow-up study

## Abstract

**Objective:**

The aim of this study was to describe the characteristics and outcomes of acute myocarditis (AM) patients while seeking accessible and valid early predictors for the development of dilated cardiomyopathy (DCM).

**Methods:**

We conducted a retrospective evaluation of 136 consecutive AM patients admitted to our hospital. The patients were categorized into two groups according to their left ventricular ejection fraction (LVEF) at presentation: those with an impaired LVEF of ≤55% and those with a normal LVEF of >55%. Multivariate logistic regression analyses were conducted to identify early predictors of DCM.

**Results:**

The median age of the study participants was 10.35 years (5.60–14.70), and most of the participants (66.91%) were males. Thirty-eight (27.94%) patients had an LVEF of ≤55%. Compared with those with an LVEF >55%, patients with an LVEF ≤55% presented significantly elevated levels of cardiac troponin I (cTnI) and N-terminal pro-B-type natriuretic peptide (NT-proBNP), as well as more pronounced clinical manifestations, including a greater prevalence of fulminant myocarditis, New York Heart Association (NYHA) class II-IV, abnormal electrocardiogram results, and enlargement of the left ventricle on echocardiography. Univariate analysis revealed that patients with an LVEF of ≤55% had an increased risk of poor outcomes and DCM development. These patients faced the greatest likelihood of death and heart transplantation within the first year following discharge. During short-term follow-up, 15.44% of the children with AM progressed to DCM. According to the multivariable analysis, a higher baseline LV end-diastolic diameter z score (LVEDD z-score) independently predicted this progression (odds ratio [OR], 2.685; 95% confidence interval [CI], 1.232–5.851; *P* = 0.013).

**Conclusions:**

Patients with AM and LVEF ≤55% had a more severe clinical course, higher rates of poor outcomes, and increased risk of DCM progression. Moreover, this subgroup was at the greatest risk for death and heart transplant within the first year post-discharge. During short-term follow-up, 15.44% of the children diagnosed with AM progressed to DCM, with a higher baseline LVEDD z-score identified as a potential early predictor for this progression.

## Introduction

Acute myocarditis (AM) is defined as inflammation of the myocardium resulting from various infectious and noninfectious causes. Its clinical manifestations are very heterogeneous, including a wide spectrum of clinical manifestations of cardiac and noncardiac insufficiency, asymptomatic or severe cardiac insufficiency, or even sudden cardiac death. Most patients with myocarditis experience a favorable prognosis, and a small proportion die or need heart transplantation (HTx) (1–4). However, chronic myocardial inflammation can lead to myocardial scarring, which subsequently triggers myocardial remodeling. This process results in ventricular dilatation, which may progress to dilated cardiomyopathy (DCM) or hypodynamic nonDCM (5). It has been documented in the literature that 6%–30% of myocarditis cases may progress to DCM, with or without a transient period of remission. Furthermore, evidence suggests that 9%–50% of DCM patients exhibit signs of myocardial inflammation (6). However, there are few clinical studies on the factors involved in the progression from AM to DCM, particularly concerning the incidence and early predictors of DCM in the short term.

The long-term survival of patients with myocarditis is closely associated with their initial presentation. Therefore, early implementation of risk stratification and management strategies is essential (7). Additionally, in AM patients, left ventricular (LV) systolic dysfunction is a predictor of poor hospitalization and long-term outcomes (8–10). Thus, patients can be risk stratified and managed according to the extent of LV systolic dysfunction (7). As a subgroup, the clinical characteristics of children with AM and LV systolic dysfunction and the associated prognosis are not well documented. Therefore, in the present study, we aimed to (1) identify early predictors of progression to DCM among patients with AM during a brief follow-up period after discharge and (2) summarize the characteristics of patients with AM and the associated prognosis in children.

## Methods

### Study population

This research involved a retrospective analysis of children who were diagnosed with AM and hospitalized at the First Affiliated Hospital of Guangxi Medical University, China, from January 2014 to October 2024. The diagnosis of myocarditis was determined according to the “Diagnostic Recommendation for Myocarditis in Children (Version 2018)” (11). The main diagnostic criteria were as follows: (1). cardiac insufficiency, cardiogenic shock or cardiocerebral syndrome; (2). cardiomegaly; (3). serum cardiac troponin T or I (cTnT or cTnI) and creatine kinase isoenzyme (CK-MB) were elevated and changed dynamically; (4). significant electrocardiographic changes (ECG or 24 h Holter ECG); and (5). cardiac magnetic resonance (CMR) imaging revealed typical myocardial inflammation. Clinically, AM implies that a short period elapses from the onset of symptoms to diagnosis (generally <1 month) (5). Therefore, only patients whose onset of symptoms occurred within 1 month before admission were included in our study. The exclusion criteria were as follows: (1) coronary artery disease; primary cardiomyopathy; a history of congenital heart disease or rheumatic heart disease; or other cardiac dysfunctions from pyemia, metabolic diseases, hyperthyroidism, poisoning, or connective tissue disease; and (2) incomplete medical records.

The final study cohort included a total of 136 patients. The patients were subsequently grouped into two categories according to their LV ejection fraction (LVEF) at presentation: (1) those with an impaired LVEF of ≤55% and (2) those with a normal LVEF of >55%. The study was approved by the institutional review board of the First Affiliated Hospital of Guangxi Medical University **(**approval number: 2025-E0303). The data were anonymized, so informed consent was not needed.

### Definition

The definition for AM progressing to DCM includes a persistence of LV end-diastolic diameter (LVEDD) z score >2 and LV systolic dysfunction (LVEF <55%) in AM patients and a longer duration of heart failure symptoms (>1 month) (5, 12). We focused on DCM following a short-term follow-up period of 1–3 months postdischarge. Additionally, the predictive factors and incidence of AM progression to DCM within this brief timeframe were determined. The diagnosis of fulminant myocarditis (FM) primarily relies on the clinical symptoms exhibited by patients. To diagnose FM, the following criteria must be satisfied: (1) sudden appearance of severe heart failure signs within 2 weeks; (2) prodromal symptoms of upper respiratory or gastrointestinal viral infections; and (3) rapid development of hemodynamic instability necessitating large doses of inotropic medications (13). The diagnosis of FM was defined as the fulminant form in this study. Left ventricular systolic dysfunction was defined as an LVEF ≤55%.

### Data collection

All clinical data, including demographics, clinical presentations, laboratory test results, ECG, echocardiography, CMR, and medical treatment data, were gathered by qualified clinicians from initial hospitalization records. To minimize the effects of extreme values, N-terminal pro-B-type natriuretic peptide (NT-proBNP) levels were log-transformed (base10). Only data from the first admission were considered for patients with more than one admission. Lab results were obtained within 24 h, whereas ECG and echocardiography data were collected within 48 h of admission. Age- and body surface area-specific Z scores were used to normalize the LVEDD. CMR imaging was performed within 3–5 days after admission and was available for 80 patients. CMR scans were performed on a 1.5 T (Magnetom Altea, Siemens Healthineers) or 3.0 T (Magnetom Prisma, Siemens Healthineers, Erlangen, Germany) clinical scanner at our center. Myocardial inflammation was diagnosed according to the 2018 Lake Louise Criteria (14). Endomyocardial biopsy (EMB) is not routinely performed at our center, and no patients underwent EMB for this study.

### Follow-up of clinical endpoints

The primary endpoint was AM progressing to DCM at the short-term follow-up (1–3 months). The second endpoint was defined as poor outcomes, including all-cause mortality and HTx. The follow-ups included regular outpatient clinic visits and telephone interviews. Follow-up ended on December 31, 2024, or at the time of death or HTx.

### Statistical analysis

Continuous variables are represented by the mean and standard deviation or by the median and interquartile range (IQR), depending on suitability (the Shapiro‒Wilk test was applied to assess the normal distribution of continuous variables). Unpaired Student's t test or the Mann‒Whitney *U*-test was used as appropriate to compare continuous variables. The Wilcoxon matched-pairs signed rank test was used to analyze paired data at different time points. Categorical variables were compared with the chi-square test or Fisher's exact test. Uni- and multivariable logistic regression analyses were performed to estimate the key independent factors predicting DCM development. Receiver operating characteristic (ROC) curves were constructed to assess the statistical significance of the numerical variables, and the Youden index was calculated to optimally determine the cutoff point for predicting DCM. For long-term survival outcomes, Kaplan‒Meier survival curves were generated, and the log-rank test was performed to compare differences in the survival rates of the two groups. The significance level for all analyses was established at *P* < 0.05.

## Results

### Baseline characteristics

The research involved 136 patients in a row. The initial clinical features of the whole study group and the two LVEF categories were determined by the LVEF at the time of admission and are presented in Table 1. The median age of the study participants was 10.35 years (5.60–14.70), and most (66.91%) were males. A total of 41 (30.14) patients presented with heart failure symptoms classified as New York Heart Association (NYHA) class II–IV, and 24 (17.65%) patients were diagnosed with FM. A total of 38 (27.94%) patients had an LVEF ≤55%, were predominantly male, and consisted mainly of older children. There was no significant difference in the demographic characteristics between the two study groups. Patients with an LVEF >55% were more likely to present with chest pain. However, heart failure symptoms and a diagnosis of FM were more common in patients with an LVEF ≤55%.

**Table 1:** Comparison based on degree of left ventricular dysfunction.

Characteristic	Total population	LVEF ≤ 55%	LVEF > 55%	*P*
*n* (%)	136 (100.00)	38 (27.94)	98 (72.06)	
Male gender, *n* (%)	91 (66.91)	27 (71.05)	64 (65.31)	0.523
Age (years)	10.35 (5.60–14.70)	12.15 (8.10–15.23)	9.60 (3.98–14.70)	0.215
Presentation, *n* (%)				
Chest pain	41 (30.15)	3 (7.89)	38 (38.78)	**0** **.** **004**
Syncope	6 (4.41)	2 (5.26)	4 (4.08)	0.672
Respiratory symptom	69 (50.74)	18 (47.37)	51 (52.04)	0.625
Gastrointestinal symptom	32 (23.53)	9 (23.68)	23 (23.47)	0.979
NYHA class II–IV	41 (30.14)	29 (76.32)	12 (12.24)	<**0**.**001**
fulminant form	24 (17.65)	11 (28.95)	13 (13.27)	**0**.**031**
Laboratory findings				
CK-MB (IU/L)	27.00 (20.00–54.00)	28.00 (19.00–58.00)	27.00 (20.00–53.00)	0.778
cTnI (ng/ml)	0.10 (0.00–2.79)	0.28 (0.05–6.14)	0.01 (0.00–2.24)	**0**.**003**
NT-proBNP (pg/ml)	311.00 (94.75–3354.00)	5324.00 (2578.50–10601.25)	204.50 (61.66–606.00)	<**0**.**001**
logBNP	2.49 (1.98–3.53)	3.73 (3.41–4.02)	2.31 (1.79–2.79)	<**0**.**001**
WBC (×109/L)	7.84 (6.35–9.99)	8.30 (6.68–10.53)	7.75 (6.26–9.98)	0.514
AST (IU/L)	37.00 (26.00–78.00)	57.00 (31.75–130.00)	34.00 (26.00–60.00)	**0**.**008**
LDH (IU/L)	286.00 (223.00–438.00)	375.50 (264.00–565.75)	261.00 (216.50–387.50)	**0**.**001**
ALB (IU/L)	40.10 (37.40–43.00)	38.65 (34.00–41.60)	41.00 (38.00–43.35)	**0**.**001**
ECG findings, *n* (%)				
Nonspecific ST-T alteration	20 (14.71)	12 (31.58)	8 (8.16)	**0**.**001**
Q waves	22 (16.18)	12 (31.58)	10 (10.20)	**0**.**002**
QT interval prolongation	29 (21.32)	9 (23.68)	20 (20.41)	0.676
ST segment elevation	35 (25.74)	8 (21.05)	27 (27.55)	0.437
LBBB	5 (3.68)	4 (10.53)	1 (1.02)	**0**.**033**
2nd,3rd AVB	17 (12.50)	3 (7.89)	14 (14.29)	**0**.**047**
PVCs	41 (30.15)	17 (44.74)	24 (24.49)	**0**.**021**
Ventricular tachycardia	13 (9.56)	9 (23.68）	4 (4.08)	**0**.**002**
Echocardiographic findings				
LVEF (%)	59.97 (±16.51)	36.79 (±12.64)	68.96(±5.11)	<**0**.**001**
LVEDD (mm)	43.34 (±11.52）	53.50 (±13.79)	39.40 (±7.48)	<**0**.**001**
LVEDD z-score	1.07 (±2.85）	4.03 (±3.52）	−0.08 (±1.33)	<**0**.**001**
Pericardial effusion, *n* (%)	31 (22.79)	18 (47.37)	13 (13.27)	<**0**.**001**
CMR findings, *n* (%)	80 (58.82)	24 (63.16)	56 (57.14)	0.522
Signs of edema (T2-based imaging)	59 (73.75)	16 (66.67)	43 (76.79)	0.346
Presence of LGE	54 (67.50)	20 (83.33)	34 (60.71)	**0**.**048**
septal invovement	21 (26.25)	15 (62.50)	6 (10.71)	<**0**.**001**
Treatment, *n* (%)				
IVIG	67 (49.26)	21 (55.26)	46 (46.94)	0.384
Steroid	81 (59.56)	25 (65.79)	56 (57.14)	0.357
Betablockers	34 (25.00)	14 (36.84)	20 (20.41)	**0**.**047**
ACEI	16 (11.76)	15 (39.47)	1 (1.02)	<**0**.**001**
Digoxin	21 (15.44)	21 (55.26)	0 (0.00)	<**0**.**001**
Diuretics	20 (14.71)	20 (52.63)	0 (0.00)	<**0**.**001**
Outcome, *n* (%)				
In-hospital mortality	2 (1.47)	2 (5.26)	0 (0.00)	0.077
DCM	21 (15.40)	21 (55.26)	0 (0.00)	<**0**.**001**

Bold values indicate statistically significant intergroup differences (*p* < 0.05).

NYHA, New York heart association; CK-MB, creatine kinase isoenzyme; cTnI, cardiac troponin I; NT-proBNP, N-terminal pro-B-type natriuretic peptide; WBC, White Blood Cells; AST, aspartate aminotransferase; LDH, lactate dehydrogenase; ALB, Albumin; ECG, electrocardiography; LBBB, left bundle branch block; AVB, atrioventricular blocks; PVCs, premature ventricular complexes; LVEF, left ventricular ejection fraction; LVEDD, left ventricular end-diastolic diameter; CMR, cardiac magnetic resonance; LGE, Late gadolinium enhancement; IVIG, intravenous immunoglobulin; ACEI, angiotensin converting enzyme inhibitor; DCM, dilated cardiomyopathy.

Patients with an LVEF ≤55% had higher admission levels of cardiac troponin I, log B-type natriuretic peptide, aspartate aminotransferase, and lactate dehydrogenase, along with lower albumin levels, compared to those with an LVEF >55%. With respect to the ECG findings, patients with an LVEF ≤55% were more likely to have abnormal Q waves and nonspecific ST-T alterations and premature ventricular complexes (PVCs) and ventricular tachycardia, as well as complete left bundle branch block (LBBB). Notably, patients with an LVEF ≤55% had higher LVEDD z-score (4.03+/−3.52 vs. −0.08 +/−1.33, *P* < 0.001) and were more likely to present with pericardial effusion than those with an LVEF >55%. In addition, CMR reports were available for only 80 (58.82%) patients in the study population.The prevalence of edema signs (T2-based imaging) in patients with an LVEF ≤55% was 66.67%, while late gadolinium enhancement (LGE) occurred in 83.33%. In patients with an LVEF >55%, edema signs were present in 76.79%, and LGE was found in 60.71%.

In terms of medical treatment, the utilization rate of intravenous immunoglobulin (IVIG) in our cohort was 49.26%, while the usage rate of steroids was 59.56%. No significant difference was observed between the two groups in the use of IVIG or steroids. Patients with an LVEF ≤55% were more likely to receive digoxin, diuretics, ACE inhibitors (ACEIs), and beta-blockers.

### Clinical outcomes

**Figure 1:**
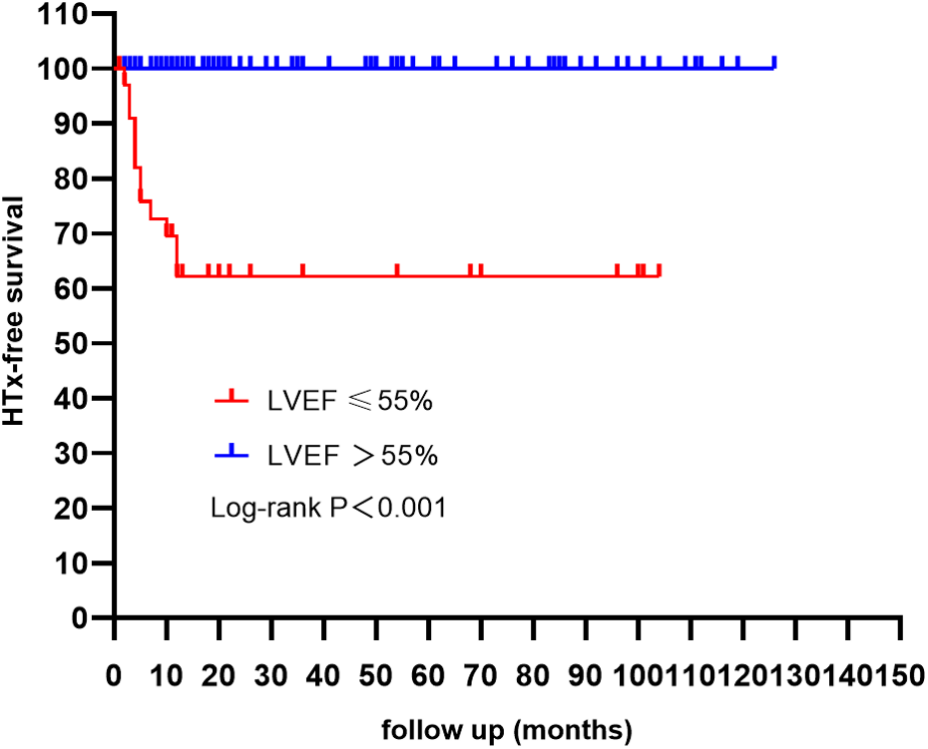
Kaplan Meier HTx-free survival curves according to LVEF. Patients with a LVEF ≤ 55% had a poor outcome when compared with those with a LVEF > 55% (log-rank test). It is noteworthy that the majority of deaths and HTx occurred within the first year following discharge. LVEF, left ventricular ejection fraction; HTx, heart transplantation.

After short-term follow-up, DCM was observed in 21 (15.44%) patients, and DCM predominantly manifested in the groups with an LVEF ≤55%. This condition constituted 55.26% of the group with an LVEF ≤55%. There was no between-group difference in in-hospital mortality. With respect to long-term outcomes, with a mean follow-up time of 17 (8–60) months, 10 (7.46%) patients died, 2 (1.49%) patients underwent HTx, and 14 (10.44%) patients were lost to follow-up. The survival free from HTx of patients with an LVEF ≤55% was markedly lower than that of patients with an LVEF >55% (Figure 1). Furthermore, among the patients with an LVEF ≤55%, all deaths or heart transplant occurred within one year of discharge.

### Subgroup analysis

**Figure 2:**
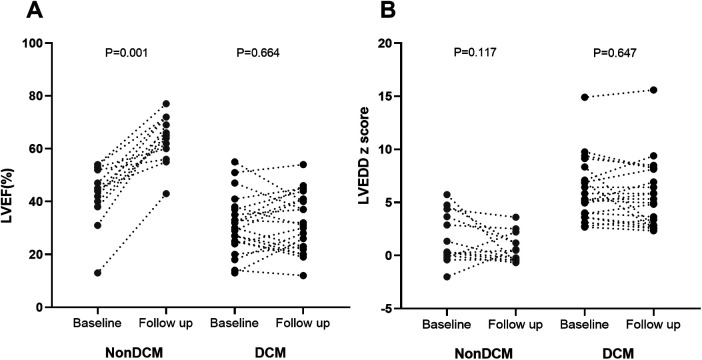
LVEF **(A)** and LVEDD z-score **(B)** at baseline and 1–3 month follow up. Wilcoxon matched-pair signed rank test was used for comparisons. LVEF, left ventricular ejection fraction; DCM, dilated cardiomyopathy; LVEDD z scores, left ventricular end-diastolic diameter z scores.

According to the short-term follow-up results, 2 hospitalized patients who died and 2 patients whose follow-up echocardiographic findings were not available were excluded. The 34 patients with an LVEF ≤55% were divided into a non-DCM group and a DCM group (Table 2). 26 cases had an LVEDD z-score > 2 at the time of diagnosis. In the DCM group, the baseline LVEF was lower but the LVEDD z-score was greater. The baseline LVEF and LVEDD z-score, as well as the follow-up LVEF and LVEDD z-score, were compared within each group (Figure 2). There was no significant change in LVEF in the DCM group, whereas the non-DCM group presented a notable increase in LVEF (*P* = 0.001). No significant alterations in the LVEDD z-scores were detected in the two groups.

**Table 2:** Comparision based on non-DCM and DCM in patients with an LVEF ≤ 55%.

Characteristic	non-DCM	DCM	*P*
*n* (%)	13 (38.24)	21 (61.76)	0.079
Gender (male)	8 (61.54)	17 (80.95)	0.254
Age (years)	10.10 (6.40–12.45)	12.80 (8.00–15.40)	0.103
Presentation, *n* (%)			
fulminant form	5 (38.46)	4 (19.05)	0.254
NYHA II–IV	8 (61.54)	11 (52.38)	0.079
Laboratory findings,			
CK-MB (IU/L)	30.00 (23.00–74.00)	23.00 (17.00–47.50)	0.103
cTnI (ng/ml)	1.95 (0.08–6.64)	0.17 (0.03–0.45)	0.096
logBNP	3.73 (3.48–3.97)	3.73 (3.34–4.07)	0.917
ECG findings, *n* (%)			
Nonspecific ST-T alteration	6 (46.15)	5 (23.81)	0.262
Q waves	3 (23.08)	9 (42.86)	0.292
2nd, 3rd AV blocks	3 (23.08)	0 (0.00)	**0** **.** **048**
LBBB	2 (15.38)	2 (9.52)	0.627
Ventricular arrhythmia	5 (38.46)	11 (52.38)	0.497
Echocardiographic findings			
Baseline LVEF (%)	45.00 (39.00–52.50)	30.00 (24.50–37.00)	**0**.**005**
Baseline LVEDD z-score	0.34 (−0.11–4.02)	5.32 (3.78–7.72)	<**0**.**001**
Baseline LVEDD z-score >2, *n* (%)	5 (38.46)	21 (100.00)	<**0**.**001**
Follow up LVEF (%)	65.00 (58.00–72.00)	31.00 (22.50–41.00)	<**0**.**001**
Follow up LVEDD z-score	0.49 (−0.44–1.70)	5.32 (2.85–8.18)	<**0**.**001**
CMR findings, *n* (%)	8 (61.54)	14 (66.67)	1.000
LLC (+)	8 (100.00)	10 (71.43)	0.254
Treatment, *n* (%)			
IVIG	9 (69.23)	11 (52.38)	0.477
Steroid	9 (69.23)	14 (66.67)	1.000
Outcome, *n* (%)			
Mortality or transplant	1 (7.70)	11 (52.38)	**0**.**011**

Bold values indicate statistically significant intergroup differences (*p* < 0.05).

NYHA, New York heart association; CK-MB, creatine kinase isoenzyme; cTnI, cardiac troponin I; BNP, pro-B-type natriuretic peptide; AST, aspartate aminotransferase; LBBB, left bundle branch blocks; LVEF, left ventricular ejection fraction; LVEDD, left ventricular end-diastolic diameter; DCM, dilated cardiomyopathy; CMR, cardiac magnetic resonance; LLC, lake louise criteria; IVIG, intravenous immunoglobulin.

### Subgroup outcomes

In the non-DCM group, the median follow-up time was 18 months (10.5–83). Two patients were lost to follow-up; one underwent HTx 6 months postdischarge due to refractory cardiac failure and pulmonary hypertension, while the other developed DCM 6 months later. Nine patients had a normalized LVEF and LVEDD.

In the DCM group, the median follow-up time was 10 months (4–24). Two patients were lost to follow-up; one underwent HTx 7 months after discharge, and ten patients died. By the end of the follow-up, five patients still presented decreased LV systolic function, whereas three patients presented normal LVEFs and LVEDDs.

### Predictors of DCM

Initially, we carried out a univariate analysis to identify significant differences between DCM patients and non-DCM patients in the entire study population. The following baseline variables were statistically significant in the univariate model: NYHA class II–IV, logBNP, Q waves, PVCs, LVEDD z score, LVEF, and pericardial effusion. We subsequently included these variables in a multivariate analysis, revealing that a greater LVEDD z score was related to an increase in the odds of DCM (odds ratio [OR], 2.685; 95% confidence interval [CI], 1.232–5.851; *P* = 0.013) (Table 3). The area under the ROC curve for the baseline LVEDD z-score in predicting DCM was 0.982 (95% CI: 0.963–1.000, *P* < 0.001), and the best cutoff value of the LVEDD z-score was 2.59 (sensitivity: 68.4%, specificity: 96.9%).

**Table 3:** Univariable and multivariable analyses for DCM in overall corhot.

Variable	Univariable analysis			Multivariable analysis		
	OR	95% CI	*P*	OR	95% CI	*P*
Male (reference:female)	0.434	0.137–1.381	0.158			
Age, per year	1.071	0.976–1.174	0.147			
Presentation						
Respiratory symptom	1.080	0.425–2.748	0.871			
Gastrointestinal symptom	0.769	0.238–2.489	0.661			
NYHA II–IV	43.225	9.310–200.685	<**0.001**	4.349	0.160–118.07	0.383
Fulminant form	1.216	0.366–4.028	0.750			
Laboratory findings						
CK-MB	1.001	0.998–1.003	0.706			
cTnI	0.959	0.877–1.049	0.361			
logBNP	5.869	2.693–12.789	<**0**.**001**	0.093	0.003–2.527	0.159
WBC	1.019	0.887–1.169	0.793			
AST	1.000	0.999–1.002	0.884			
LDH	1.001	1.000–1.001	0.178			
ALB	0.955	0.884–1.032	0.243			
ECG findings						
Nonspecific ST-T alteration	2.165	0.686–6.838	0.188			
Q waves	5.654	1.998–15.996	**0**.**011**	1.498	0.098–22.877	0.772
QT interval prolongation	1.618	0.563–4.65	0.371			
ST segment elevation	0.884	0.297–2.628	0.824			
LBBB	3.789	0.593–24.208	0.159			
PVCs	3.110	1.196–8.086	**0**.**020**	2.248	0.117–43.100	0.591
Ventricular tachycardia	3.496	0.923–13.233	0.065			
Echocardiographic findings						
LVEDD z-score	3.685	2.077–6.539	<**0**.**001**	2.685	1.232–5.851	**0**.**013**
LVEF	0.852	0.802–0.906	<**0**.**001**	0.857	0.721–1.019	0.081
Pericardial effusion	5.326	1.982–14.314	**0**.**001**	25.971	0.356–1895.159	0.137
IVIG	0.893	0.351–2.271	0.812			
Steroid	1.415	0.530–3.782	0.488			

Bold values indicate statistically significant intergroup differences (*p* < 0.05).

DCM, dilated cardiomyopathy; CK-MB, creatine kinase isoenzyme; cTnI, cardiac troponin I; NT-proBNP, N-terminal pro-B-type natriuretic peptide; WBC, white blood cells; AST, aspartate aminotransferase; LDH, lactate dehydrogenase; ALB, Albumin; ECG, electrocardiography; LBBB, left bundle branch blocks; PVCs, premature ventricular complexes; LVEF, left ventricular ejection fraction; LVEDD, left ventricular end-diastolic dimension; IVIG, intravenous immunoglobulin; CI, confidence interval; OR, odds ratio.

## Discussion

In this retrospective study, we conducted a systematic analysis of the clinical features and outcomes of children diagnosed with AM. We evaluated the incidence and identified potential predictors of DCM. This subgroup with an LVEF ≤55% made up 27.94% of the total cohort and exhibited significantly greater rates of poor outcomes and DCM development than the group with a normal LVEF did. Furthermore, patients with an LVEF ≤55% were at the highest risk of death and HTx within the first year after discharge. During short-term follow-up (1–3 months), our findings revealed that 15.44% of children with AM progressed to DCM. A higher baseline LVEDD z-score was identified as a potential early predictor of AM-to-DCM progression.

In the entire cohort, the majority of the children were male, a finding consistent with previous multicenter studies (9, 15, 16). Notably, only 27.94% of our patients had an LVEF ≤55% (predominantly male, median age 12.2 years), contrasting with another study reporting 45.12% with moderate-to-severe LV systolic dysfunction (more females, younger age) (9). These demographic differences may primarily reflect varying inclusion/exclusion criteria. Additionally, the heterogeneous clinical manifestations of pediatric myocarditis pose significant diagnostic challenges, potentially introducing selection bias.

In our study, the overall cohort demonstrated a benign prognosis. As expected, most poor outcomes were observed in patients with an LVEF ≤55%, these findings are in accordance with those of previous studies (9, 10). Our results also align with prior research showing that the risk of death was highest within the first year post-discharge (17, 18). Interestingly, an adult study reported most adverse events in patients with LVEF <50%, but no deaths or HTx occurred within 1 year post-diagnosis (10). This mortality pattern contrasts sharply with our findings, possibly due to differences in diagnostic criteria and study populations. The results of a nationwide study conducted in Australia indicate that children are at the highest risk of mortality within the first year following the diagnosis of DCM (19). Our subgroup analysis revealed that all deceased patients fulfilled the diagnostic criteria for DCM during short-term follow-up, suggesting that early progression to DCM is associated with a worse long-term prognosis. Therefore, these patients should be managed in accordance with established guidelines for the diagnosis and treatment of DCM.

Our study revealed a 15.44% short-term progression rate to DCM in children with AM. Assessing the incidence of AM progressing to DCM remains challenging because of its complex clinical course. The transition from AM to DCM can occur in the short term, over a period of months, or in the medium to long term, over a period of years, potentially including periods of remission. Some researchers have hypothesized that certain cases of idiopathic DCM in adults might originate from unrecognized myocarditis during childhood (20). Clinical studies examining risk factors for the transition from AM to DCM remain limited and are often subject to inconsistent endpoint definitions. Krasic, S. et al. (21) reported that the incidence of DCM was 17.70%, with the highest risk observed in patients with an LVEF between 40% and 50%. Additionally, acute FM was shown to be an independent predictor of DCM development. Rodriguez-Gonzalez, M. et al. (8) reported that an LVEF < 30% was an independent predictor of death, heart transplant, or persistent LV systolic dysfunction or dilation. The study by Kim, G. et al. (18) demonstrated that a higher LVEDD z-score significantly predicts adverse outcomes, including mortality and incomplete recovery. Similarly, a study conducted in adults concluded that LVEDD was an independent risk factor for persistent LV systolic dysfunction (22).

The most important relevant result of our study is that the baseline LVEDD z-score was a potential early predictor for DCM. According to our subgroup analyses, the baseline LVEF significantly improved in the non-DCM group, whereas no such improvement was observed in the DCM group. A study conducted in adults concluded that early improvement in LVEF was independently associated with favorable long-term outcomes (23). These observations indicate the potential for short-term recovery of LVEF in patients presenting with AM and reduced LVEF, but our study further revealed that patients with elevated LVEDD z-score at presentation did not show potential for short-term recovery. Recent-onset myocarditis (with symptom duration <30 days), including fulminant myocarditis, typically does not exhibit significant ventricular enlargement (24–26). Inflammation-mediated ventricular dilatation may occur gradually over time. In an animal model of coxsackievirus B3-induced myocarditis, significant LV dilatation was observed 28 days post-infection (27). In our study, a small subset of patients presenting with marked LV enlargement at initial diagnosis likely experienced a prolonged inflammatory process. Due to insufficient attention to mild symptoms in children, a discrepancy may exist between the presumed and actual times of clinical onset, representing a common challenge in the clinical diagnosis of pediatric myocarditis. We investigated early predictors of the DCM phenotype following the acute phase of clinically diagnosed acute myocarditis (1 to 3 months post-discharge). Our findings suggest that in cases of myocarditis with LVEF ≤ 55%, LV enlargement does not resolve within a short timeframe, possibly due to chronic inflammation-mediated ventricular remodeling. This may explain why elevated LVEDD-z score at initial diagnosis serves as potential early predictor for DCM. These findings underscore the importance of monitoring not only LVEF changes but also LVEDD z-score alterations.

## Conclusions

Patients with AM and LVEF ≤55% had a more severe clinical course, higher rates of poor outcomes, and increased risk of DCM progression. Moreover, this subgroup was at the greatest risk for death and HTx within the first year post-discharge. During short-term follow-up, 15.44% of the children diagnosed with AM progressed to DCM, with a higher baseline LVEDD z-score identified as a potential early predictor for this progression. However, these findings should be interpreted cautiously due to the short-term observational nature of our study; longer-term follow-up is needed to validate these preliminary associations. Early risk stratification is critical in pediatric myocarditis management. All AM patients with reduced LVEF require long-term follow-up to monitor for adverse outcomes.

## Limitations

This is a single-center retrospective study that has intrinsic methodological limitations. Furthermore, the study participants were recruited from tertiary referral centers, which introduces selection bias. The study spans 10 years, and a small number of patients were enrolled based on clinical diagnosis without EMB-proven myocarditis. CMR was performed in only a subset of patients, leading to a reduction in statistical power and potentially failing to reflect the true scenario. We evaluated only the short-term incidence and risk factors for progression to DCM due to the unavailability of long-term follow-up echocardiographic data. Our findings are based solely on short-term follow-up data and therefore should not be extrapolated to predict long-term outcomes. Nonetheless, we assert that our research captures the real-world outcomes of children with clinically diagnosed AM. Future research should include multicenter, prospective studies to further validate our findings.

## Data Availability

The raw data supporting the conclusions of this article will be made available by the authors, without undue reservation.
